# An abundant ‘*Candidatus* Phytoplasma solani’ tuf b strain is associated with grapevine, stinging nettle and *Hyalesthes obsoletus*

**DOI:** 10.1007/s10658-014-0455-0

**Published:** 2014-06-04

**Authors:** A. Aryan, G. Brader, J. Mörtel, M. Pastar, M. Riedle-Bauer

**Affiliations:** 1 Austrian Institute of Technology, Konrad-Lorenz-Straße 24, 3430 Tulln, Austria; 2 Höhere Bundeslehranstalt und Bundesamt für Wein- und Obstbau Klosterneuburg, Wienerstraße 74, 3400 Klosterneuburg, Austria

**Keywords:** Bois noir, Stolbur, *Stamp*, *vmp1*, *secY*, ‘*Candidatus* phytoplasma convolvuli’

## Abstract

Bois noir (BN) associated with ‘*Candidatus* Phytoplasma solani’ (Stolbur) is regularly found in Austrian vine growing regions. Investigations between 2003 and 2008 indicated sporadic presence of the confirmed disease vector *Hyalesthes obsoletus* and frequent infections of bindweed and grapevine. Infections of nettles were rare. In contrast present investigations revealed a mass occurrence of *H. obsoletus* almost exclusively on stinging nettle. The high population densities of *H. obsoletus* on *Urtica dioica* were accompanied by frequent occurrence of ‘*Ca.* P. solani’ in nettles and planthoppers. Sequence analysis of the molecular markers *secY, stamp, tuf* and *vmp1* of stolbur revealed a single genotype named CPsM4_At1 in stinging nettles and more than 64 and 90 % abundance in grapevine and *H. obsoletus,* respectively. Interestingly, this genotype showed tuf b type restriction pattern previously attributed to bindweed associated ‘*Ca.* P. solani’ strains, but a different sequence assigned as tuf b2 compared to reference tuf b strains. All other marker genes of CPsM4_At1 clustered with tuf a and nettle derived genotypes verifying distinct nettle phytoplasma genotypes. Transmission experiments with *H. obsoletus* and *Anaceratagallia ribauti* resulted in successful transmission of five different strains including the major genotype to *Catharanthus roseus* and in transmission of the major genotype to *U. dioica*. Altogether, five nettle and nine bindweed associated genotypes were described. Bindweed types were verified in 34 % of grapevine samples, in few positive *Reptalus panzeri,* rarely in bindweeds and occasionally in *Catharanthus roseus* infected by *H. obsoletus* or *A. ribauti*. ‘*Candidatus* Phytoplasma convolvuli‘(bindweed yellows) was ascertained in nettle and bindweed samples.

## Introduction

Phytoplasmas are small, wall-less mollicutes causing more than 700 diseases in hundreds of plant species. Within plants they colonize the phloem and are transmitted by phloem feeding insects such as leafhoppers, planthoppers and psyllids (Weintraub and Beanland 2006; Bertaccini and Duduk 2009). *‘Candidatus* Phytoplasma solani’ (Quaglino et al. 2013) formerly known as Stolbur phytoplasma (taxonomic group 16SrXII-A) affects a wide range of wild and cultivated plants including grapevines, where it induces Bois noir (BN), a disease widespread in Europe and the Mediterranean area (COST action FA 0807 2014). The spread of BN occurs via a disease cycle including herbaceous host plants as phytoplasma reservoirs and insect vectors. *Urtica dioica* and *Convolvulus arvensis* are considered to be the main phytoplasma sources with the disease transmitted by Auchenorrhyncha species. Within this group all confirmed natural BN vectors are planthoppers belonging to the family Cixiidae (Hemiptera). *Hyalesthes obsoletus* is regarded as main vector in many countries (Maixner et al. 1995, Maixner 2011). *C. arvensis* and *U. dioica* are among other plant species known as hosts for nymphs and adults of *H. obsoletus* (Holzinger et al. 2003) from which they take up the phytoplasmas. Occasional feeding of the insects on grapevines results in transmission of the pathogen, and subsequently in BN (Langer and Maixner 2004; Maixner 2011). Recently it has been demonstrated that also the Cixiidae *Reptalus panzeri* can transmit BN to grapevine seedlings in South Eastern Europe (Cvrković et al. 2014). Transmission experiments with several Cicadellidae (Hemiptera, Auchenorrhyncha) species have revealed that the leafhopper *Anaceratagallia ribauti* transmits stolbur phytoplasmas to *Vicia faba* (Riedle-Bauer et al. 2008). Up to now, however, the transmission to grapevine has not been proven (Riedle-Bauer unpublished).

On the basis of the elongation factor *Tu* (*tuf*) gene sequences ‘*Ca.* P. solani’ strains are attributed to two main genetic types, tuf a and tuf b. It is presumed that these tuf types are linked to different natural epidemic cycles of stolbur phytoplasma in the field. In Germany, tuf a strains of ‘*Ca*. Phytoplasma solani’ spread via an epidemic cycle including *U. dioica* as main herbaceous host and tuf b phytoplasmas predominantly via a cycle with *C. arvensis* (Langer and Maixner 2004, Johannesen et al. 2012). Tuf b phytoplasmas are also found in a number of other weedy plants (Riedle-Bauer et al. 2006; Johannesen et al. 2012; Cvrković et al. 2014).

In addition to the analysis of the *tuf* gene, other genes namely *secY,* encoding for a major membrane unit of the secretory pathway, *vmp1* and *stamp* were used to characterise the genetic diversity of ‘*Ca.* P. solani’ in the Euro-Mediterranean area in multilocus sequencing approaches (Cimerman et al. 2009; Fabre et al. 2011A; Pacifico et al. 2009). The latter two encode membrane proteins and were shown to have higher sequence variability than *secY* and *tuf* (Cimerman et al. 2009, Fabre et al. 2011A; 2011B, Fialová et al. 2009, Johannesen et al. 2012). *Stamp* encodes the ‘*Ca*. P. solani’ ortholog of the antigenic membrane protein AMP of *‘Ca.* P. asteris’, which specifically recognizes the actin microfilament of its leafhopper vectoring species (Suzuki et al. 2006). As shown in ‘*Ca*. P. asteris’, ‘*Ca*. P. solani’ *Stamp* gene is submitted to a positive selection pressure indicating interactions of the gene with the phytoplasma hosts (Fabre et al. 2011A). Consequently, the *stamp* characterisation based on the sequence data also allows a more fine-tuned differentiation of ‘*Ca.* P. solani’ genotypes. Four *stamp* subclusters found in maximum parsimony (MP) analysis corresponding to a single tuf a cluster and three subclusters within tuf b clusters all in all more than 25 genotypes have been described (Fabre et al. 2011B; Cvrković et al. 2014). Moreover, tuf a genotypes are localised in a single monophyletic cluster after analysis of the *vmp1* gene, while tuf b types are polyphyletic in *vmp1* analysis (Johannesen et al. 2012).

In Austria BN is widespread in the all vine growing regions. Investigations between 2003 and 2008 frequently ascertained ‘*Ca.* P. solani’ in *C. arvensis* and grapevines, whereas infections of *U. dioica* were not common and restricted to the very South (Styria). In most parts of the country *H.obsoletus* was rare or not detectable. Only in southern Styria significant population densities of *H.obsoletus* were ascertained. The insects were solely collected on *C. arvensis*, *U. dioica* was never found colonised. Analysis of tuf-types by PCR/RFLP showed exclusive presence of tuf type b (Riedle-Bauer et al. 2006, Riedle-Bauer et al. 2008, Tiefenbrunner et al. 2007, Sára and Riedle-Bauer 2009).

BN is a disease characterized by sudden outbreaks and subsequent decreases (Maixner, 2011). Recently Šafářova and co-workers (Šafářova et al. 2011) observed a dramatic rise of *H.obsoletus* populations in South Moravia close to Austria. So the aim of the present study was to collect updated data on the epidemiology of BN in Austria and to characterise ‘*Ca.* P. solani’ strains present in and around vineyards in Austria by sequence analysis of molecular markers including *stamp, secY, tuf* and *vmp1*.

## Material and methods

All plant and insect samples included in the present study were taken between September 2011 and September 2013.

Sampling of *H. obsoletus* was carried out in 30 vineyards and their surroundings in Eastern Austria (in the parts of the country where grapevines are cultivated) (Table 1). *A. ribauti* was collected at 4 test sites (Langenzersdorf, Klosterneuburg, Langenlois, Thürnthal), *R. panzeri* at one test site (Falkenstein 2). Insects were collected by vacuum sampling directly from *U. dioica* and *C. arvensis* using a modified garden blower-vac (Stihl, Dieburg, Germany). In case of *H. obsoletus* caught individuals were counted with reference to the size of the analysed nettle or bindweed plot. This allowed a rough estimation of insect numbers per m^2^ of ground cover. At the locations Falkenstein 1–3, Einöd/Kitzeck 1 and 2 and Rust 1–4 estimation of population size was based on 3–4 samplings per year in 2012 and in 2013. At all other locations population densities were surveyed only once per year.

**Table 1:** Population density of H. obsoletus at test locations all over Eastern Austria

Location	Federal Province	Population density of *H. obsoletus* on *U. dioica*	Population density of *H. obsoletus* on *C. arvensis*
Althöflein	NÖ	2	–
Blumenthal	NÖ	2	–
Falkenstein 1	NÖ	3	0
Falkenstein 2	NÖ	3	0
Falkenstein 3	NÖ	0	0
Gaiselberg	NÖ	0	0
Hagenbrunn	NÖ	2	–
Herrenbaumgarten	NÖ	2	0
Hohenruppersdorf	NÖ	0	0
Klein Schweinbarth	NÖ	3	0
Klosterneuburg	NÖ	0	2
Langenlois	NÖ	2	0
Langenzersdorf	NÖ	0	0
Niedersulz 1	NÖ	2	0
Niedersulz 2	NÖ	2	–
Thürnthal/Fels am Wagram	NÖ	2	–
Schrattenberg	NÖ	0	0
Zistersdorf	NÖ	2	0
Deutsch Haseldorf/Klöch	STMK	0	0
Kitzeck	STMK	2	0
Einöd/Kitzeck 1	STMK	3	Not assignable
Einöd/Kitzeck 2	STMK	3	–
Rust 1	BGLD	3	Not assignable
Rust 2	BGLD	3	Not assignable
Rust 3	BGLD	3	0
Rust 4	BGLD	3	–
St. Margarethen	BGLD	0	–
Groß Höflein 1	BGLD	2	0
Groß Höflein 2	BGLD	2	0
Groß Höflein 3	BGLD	2	–

- not analysed

0 no *H. obsoletus* detected

1 single individuals

2 less than 10 individuals per m^2^ of ground cover

3 more than 10 individuals per m^2^ of ground cover

Not assignable: Due to frequent occurrence of *H. obsoletus* on *U. dioica* and ground covers with both U. dioica and C. arvensis clear assignment to host for egg laying and larval development not possible

NÖ- Lower Austria, STMK- Styria, BGLD-Burgenland

Plant samples (*Vitis vinifera, U. dioica, and C. arvensis*) were taken in and around diseased vineyards and from the weeds on which infected insects had been collected. Field trapped *H. obsoletus*, *R. panzeri* and *A. ribauti* were used for transmission trials with *Catharanthus roseus* (cv. “Sorbas Reinweiß”, Austrosaat, Vienna, Austria)*, U. dioica and Convolvulus arvensis. Catharanthus roseus* and *Convolvulus arvensis* (field collection) were grown from seeds and submitted to transmission experiments after around 2–3 months (length 8–10 cm). *U. dioica* plants were collected in an Alpine region where *Hyalesthes* and *Reptalus* species are not known to occur. All nettles were tested negative by nested PCR. In general, transmission trials were carried out with 10 to 50 insects per experiment. In case of *R. panzeri*, however, catches were low, so only two experiments, one with eight and one with four specimens were conducted. The insects were transferred to single test plants, covered by cylindrical cages (diameter 9 cm, height 25 cm) and kept in a growth chamber at 23 °C under long day (L16:D8) conditions (Riedle-Bauer et al. 2008). 6–16 weeks later the test plants were inspected visually and sampled for PCR analysis.

DNA extraction from plants and insects was carried out as published earlier (Maixner et al. 1995, Langer and Maixner, 2004). Insects from all locations listed in Table 1 except Blumenthal, Einöd/Kitzeck 1, Langenlois and Thürnthal were examined. For analysis of *V. vinifera, Catharanthus roseus,* and *Convolvulus arvensis* leaf samples were processed. In case of *U. dioica* both leaves and roots were extracted separately. Presence of ‘*Ca*. P. solani’ was surveyed by nested PCR procedures with primers P1/P7 (Deng and Hiruki 1991) and STOLF/STOLR (Maixner et al. 1995), with fTUF1/rTUF1 and fTUFAY/rTUFAY (*tuf*; Schneider et al. 1997) as well as with POSecR1/POSecF1 and POSecF3/POSecR3 (*secY*; Fialová et al., 2009). TUFAY fragments were further investigated by RFLP as proposed by Langer and Maixner (2004). POSec3 amplicons were analysed by RFLP with Hinf1 (New England Biolabs, Ipswich, USA) according to the manufacturer’s instructions. Hinf1 restriction products were separated by electrophoresis on 4.5 % (w/v) polyacrylamide gels, stained with ethidium bromide and visualized on a UV transilluminator. For sequence analysis, phytoplasma DNA was amplified directly as previously described with the primer pairs Stamp fw-0 and rv-0 (Fabre et al., 2011A), fTUFAY and rTUFAY, POSecF3 and POSecR3, TYPH10F and TYPH10R (*vmp1*, Fialová et al., 2009), and for 16S with 5-CTAATACATGCAAGTCGAACG-3 (R16mF2m) and 5- TGACGGGCGGTGTGTACAAACC-3 (R16R2m) for 40 cycles, 30′ 94C, 45′ 58 °C and 90′ at 72 °C with 10 min final extension (modified from Lee et al., 1998). The PCR products were sequenced using the primers PosecR3, Stamp fw-0, TYPH10F, TYPH10R, fTUFAY, rTUFAY or R16R2m. Sequences were deposited in NCBI under the accession numbers shown in Figs. 5 and 6: KJ469710 (*tuf* bindweed yellows) and KJ469736 (16 s bindweed yellows). Sequences were analysed in BioEdit (Hall, 1999), aligned with ClustalW2 (UPGMA Clustering). Trees and the phylogenetic relationships were constructed with MP and Maximum Likelihood (ML) with PHYML (Guindon and Gascuel, 2003) on the T-rex software platform (Boc et al. 2012). ML was performed with the HKY85 substitution model without gamma distribution and branch support with 2000 bootstrap replicates.

## Results

### Occurrence of *Hyalesthes obsoletus*

Investigations between 2003 and 2008 showed no presence of *H.obsoletus* on *U. dioica*. The current inventory revealed significant numbers of *H. obsoletus* at 22 out of 30 test sites on that plant species*.* Very high insect densities exceeding 10 individuals per m^2^ of ground cover allowing catches of hundreds of individuals occurred at many sites in all investigated provinces. Insects feeding on *Convolvulus arvensis* were observed at four test sites. Only at one of these sites, however, the *H. obsoletus* population was clearly assignable to *Convolvulus arvensis*. At the other three sites ground covers with both *U. dioica* and *Convolvulus arvensis* and high insect densities on *U. dioica* made a clear assignment to a host for egg laying and larval development impossible (Table 1).

### Presence of phytoplasmas in insects and weeds

**Fig. 1:**
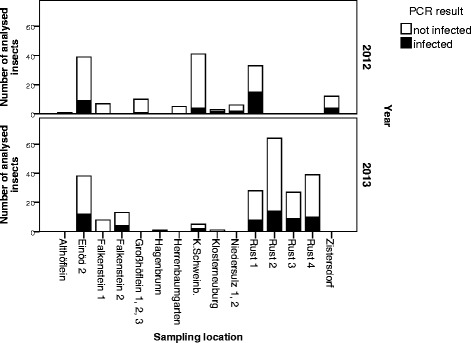
Infection rates of *H. obsoletus* at selected investigation sites listed in Table 1 in the Austrian vine growing Federal Provinces of Burgenland (Großhöflein, Rust1-4), Styria (Einöd) and Lower Austria (all other locations). Individuals from Großhöflein 1–3 and Niedersulz 1 and 2 were pooled

PCR analyses showed frequent presence of ‘*Ca*. P. solani’ in *H. obsoletus.* In total 96 out of 374 specimens, all of them collected on *U. dioica*, were found infected. Phytoplasma bearing insects were observed in all investigated Federal Provinces of Austria. Infection rates observed in 2012 and 2013 are illustrated in Fig. 1. The phytoplasmas were also ascertained in two out of 8 *R. panzeri* individuals sampled on nettles in Falkenstein. No phytoplasmas were detected in 45 *A. ribauti* individuals collected on bindweeds in Langenzersdorf and Klosterneuburg.

**Fig. 2:**
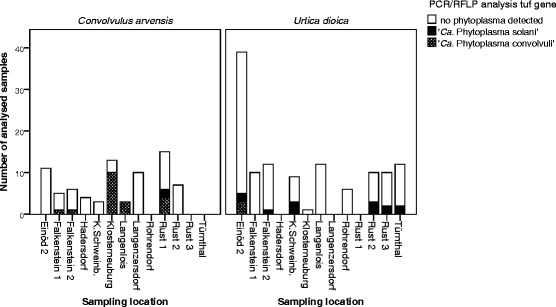
Infection rates of weed samples at several investigation sites in the Austrian vine growing provinces of Burgenland (Rust1-3), Styria (Einöd) and Lower Austria (all other locations)

PCR amplification of the *tuf* gene followed by RFLP analysis with HpaII of stinging nettles allowed the detection of ‘*Ca*. P. solani’ in 13 out of 121 samples. (Fig. 2). Infected nettles originated from six locations in all Federal Provinces. In three samples collected in Einöd/Kitzeck the *tuf* RFLP pattern did not match previously described ‘*Ca.* P. solani’ *tuf* patterns. These samples were infected with ‘*Ca*. P. convovuli’ as indicated by identical sequence to the16S ribosomal RNA of a bindweed-yellows strain from Italy (BY-I1016) sequenced with R16R2m primers (Martini et al. 2012). Only 2 ‘*Ca*. P. solani’ infections were observed in the 90 bindweed samples collected at 10 locations. More frequently, however, bindweed samples were found infected with ‘*Ca*. P. convolvuli’ (Fig. 2). ‘*Ca*. P. convolvuli’ infected bindweeds showed visual disease symptoms in the form of stunting and yellowing.

### Transmission experiments

**Fig. 3:**
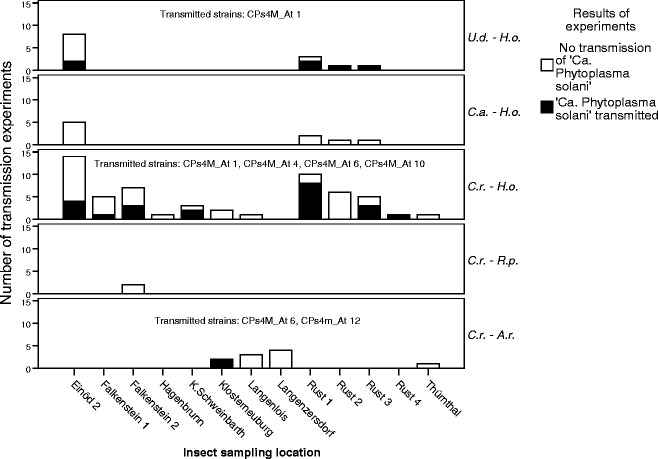
Results of transmission experiments. Transmissions were performed with 10–50 field collected insects per trial in cylindrical cages as described in material and methods. U.d. – H.o. Experimental transmission to *U. dioica* by *H. obsoletus*. C.a. – H. o. Experimental transmission to *Convolvulus arvensis* by *H. obsoletus*; C.r. – H. o. Experimental transmission to *Catharanthus roseus* by *H. obsoletus*; C.r. – R.p. Experimental transmission to *Catharanthus roseus* by *R. panzeri*; C.r. – A. r. Experimental transmission to *Catharanthus roseus* by *A. ribauti*

The transmission experiments conducted during this study frequently led to ‘*Ca*. P. solani’ infected test plants. *H. obsoletus* transmitted the phytoplasma to *Catharanthus roseus* in 22 out of 56 experiments and to *U. dioica* in 6 out of 13 experiments. Transmissions by *H.obsoletus* to *Convolvulus arvensis* were not observed. *A. ribauti* vectored the phytoplasma to *Catharanthus roseus* in two cases. No positive transmission trials were observed in case of *R. panzeri* (Fig. 3).

### ‘*Ca*. P. solani’ types

**Fig. 4:**
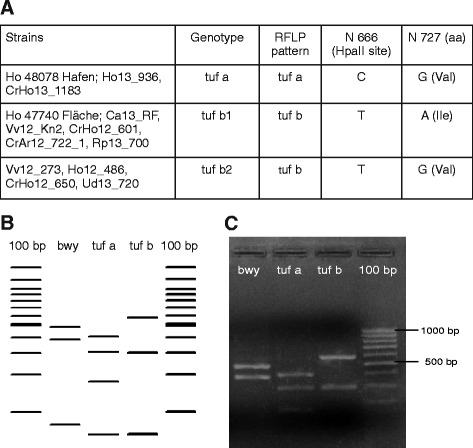
Analysis of the TUF-AY fragments of the elongation factor Tu (tuf) gene. **a** ‘*Ca*. P. solani’ types found in this study. Genotypically, 3 different sequences have been detected (tuf a, tuf b1 and tuf b2) corresponding to two HpaII types (tuf a and tuf b). Sequence differences are indicated for nucleotide (N) position after the Tuf start codon. Tuf a and tuf b reference strains from Germany and strains identified in this study (one example from each detected host species) are indicated. Vv: grapevine. Ca: bindweed. Ud: stinging nettle. Cr *Catharanthus roseus;* Ho *H. obsoletus*; Ar *A. ribaut*i; Rp *R. panzeri*, aa: amino acid. Virtual (**b**) and sample (**c**) gel showing patterns of TUFAY fragments of a bindweed yellows (bwy) strain and of ‘*Ca*. P. solani’ tuf a and tuf b digested with HpaII

Positive samples were analysed with the *tuf* gene primers and the resulting TUFAY fragments were further characterized by RFLP with HpaII. All ‘*Ca.* P. solani’ positive nettle and grapevine samples collected in this study showed a restriction identical to tuf b references from Germany (47740, *H. obsoletus* Flache and 47629, *H. obsoletus* Pfalzgraben). Seventy-seven infected *H. obsoletus* and the 22 infected *Catharanthus roseus* resulting from transmission experiments with this insect species were analysed using the same method. 95 % of these samples showed a tuf b pattern, while the remaining revealed a pattern identical to German tuf a references (48061, *H. obsoletus* Kesten and 48078, *H. obsoletus* Hafen). To confirm these results sequencing of the TUFAY fragments was performed. This allowed a discrimination of three *tuf* sequence types: identical to tuf a references 48061 and 48078 (in the following tuf a), identical to tuf b references 47629 and 47740 (in the following named tuf b1), or with a nucleotide substitution to both sequences, but *tuf* b restriction pattern (named tuf b2) (Fig. 4a). Tuf a corresponded to 5 % of the *H. obsoletus* *tuf* fragments, tuf b1 to 4 % of the *H. obsoletus* *tuf* sequences (these 4 % corresponded all to *Catharanthus roseus* infected by *H. obsoletus*) and to 34 % of the *tuf* positive grapevines. Interestingly, all *tuf* sequences derived from nettle, 91 % of the *tuf* sequences from *H. obsoletus* (all infected insects collected from nettle) and the remaining 66 % of the grapevine *tuf* sequences matched tuf b2. Although tuf b2 showed a tuf b RFLP, it was intermediate between tuf a and b references in the *tuf* polymorphic sites: A “T” was found in position 666 (after the Start) as in the reference tuf b samples causing a tuf b RFLP, while position 727 consisted of a “G” as in tuf a reference samples (Fig. 4a). Analysis of the two infected *Catharanthus roseus* plants obtained from transmission experiments with *A. ribauti* showed a tuf b restriction pattern and a tuf b1 sequence.

fTUFAY and rTUFAY primers also amplified a product from the bindweed yellows positive nettle and bindweed samples. The resulting fragment had 89 % homology to stolbur *tuf* sequences and has been deposited in the genebank (accession number KJ469710). Fig. 4b and c show a virtual and an exemplified HpaII restriction analysis from bindweed yellows and tuf a and tuf b stolbur types, allowing a fast identification of this phytoplasma also with *tuf* RFLP analysis.

**Fig. 5:**
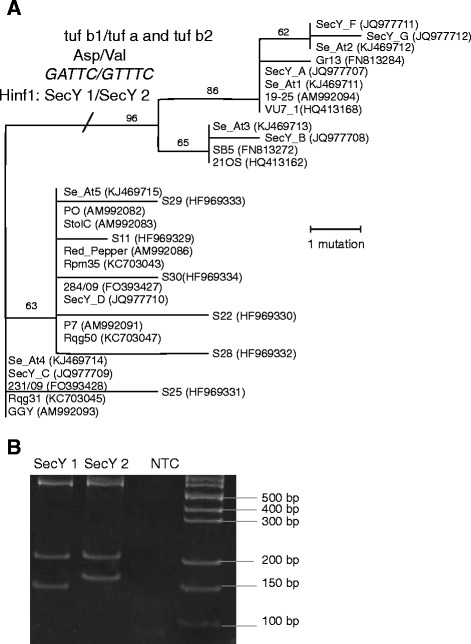
*SecY* analysis showed a clear separation in two distinct groups containing a Hinf1 site allowing RFLP analysis. **a** Phylogenetic *secY* consensus tree of PosecF3/PosecR3 fragments and corresponding database entries (reference sequences from Cimerman et al. 2009; Cvrković et al. 2014; Fabre et al. 2011A; Johannesen et al. 2012; Mitrovic et al. 2014; Semetey et al. 2999 unpublished; Seruga-Music et al. 2011) analysed by Maximum Likelihood. The numbers above the branches indicate the bootstrap values >60 (2,000 replicates). Differential Hinf1 site resulting in a corresponding amino acid substitution and 2 *secY* restriction types is indicated. **b** RFLP pattern obtained after digestion of PosecF3/PosecR3 fragments with Hinf1; SecY 1 corresponds to the “classical” tuf b1 type, SecY 2 to the tuf b2 type, NTC no template control. Tuf a strains were identical to tuf b2 strains in this analysis

To further characterise the ‘*Ca.* P. solani’ strains occurring in Austria we performed multilocus sequencing with *secY, stamp* and *vmp1* (Cimerman et al., 2009; Fabre et al., 2011A). The majority of *secY* sequences belonged to a genotype we referred to as Se_At1. All sequences matching the tuf b2 type had this *secY* genotype. It was ascertained in the majority of the *H. obsoletus* and grapevines samples and in all nettles. Tuf a strains were only found in *H. obsoletus* and *H. obsoletus-*infected *Catharanthus roseus* and were either corresponding to Se_At1, Se_At2 or Se_At3. Se_At5 always correlated with tuf b1 and was found in a third of the grapevine samples and in a few *H. obsoletus, H. obsoletus* infected *Catharanthus roseus*, in *R. panzeri* and in *A. ribauti*- infected *Catharanthus roseus*. Se_At4 also associated with tuf b1 was found in a single grapevine sample from Klosterneuburg. Interestingly, all Austrian strains with Se_At1-3 on the one hand (nettle) and Se_At4-5 (bindweed) on the other hand were clearly separated in the tree of sequences analyzed by maximum of likelihood (ML). The same pattern was valid for the bindweed/nettle-defined strains of the databases. The two types were also distinguishable by Hinf1 RFLP analysis resulting in pattern SecY 1 corresponding to tuf b1 types and SecY 2 corresponding to tuf a and tuf b2 types (Fig. 5).

**Fig. 6:**
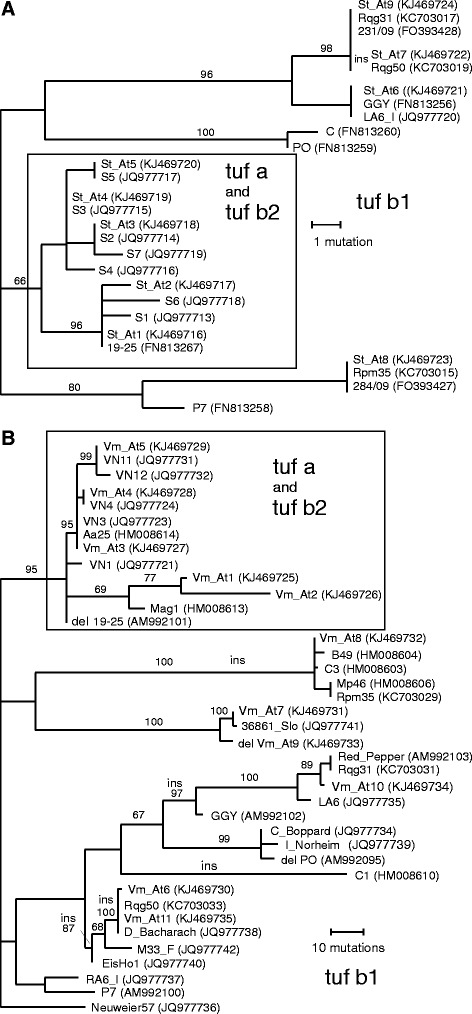
**a** Phylogenetic *stamp* tree of Stamp fw-0 and rv-0 fragments and **b** *vmp1* tree of TYPH10F and TYPH10R fragments as well as corresponding database entries (reference sequences from Cimerman et al, 2009; Cvrković et al. 2014; Fabre et al., 2011A; Johannesen et al., 2012; Mitrovic et al., 2014; Murolo et al., 2010; 2013) were analysed by Maximum Likelihood. The numbers above the branches indicate the bootstrap values >60 (2,000 replicates). Insertions (ins) and deletions (del) are indicated. Vm_At9, PO and 19–25 contain large deletions in the region of the repeated domain B (Cimerman et al, 2009). Corresponding tuf a and tuf b2 clusters are indicated

The more variable genes *stamp* and *vmp1* encoding for surface proteins showed further differentiation. In our collection, we distinguished nine *stamp* types (St_At1-9) and 11 *vmp1* types (Vm_At1-11). St_At1 was by far the most prevalent type (Table 2) and was found in all investigated ‘*Ca.* P. solani’ positive stinging nettle samples and in 64 % of the positive grapevines. Moreover, 91 % of the positive *H. obsoletus* samples contained this *stamp* genotype. St_At1 was not found in *Convolvulus arvensis,* in *R. panzeri* and in *A. ribauti* or *Catharanthus roseus* infected by *A. ribauti*. The St_At1 genotype of our collection was linked, without exception, to the *vmp* type Vm_At1, to Se_At1 and tuf b2. St_At2 could be discriminated from St_At1 by a single bp exchange. This sequence was found in a single grapevine plant in Burgenland and was linked to Vm_At2. St_At3 and St_At5 linked to Vm_At3 and Vm_At5, respectively, were found in one *H. obsoletus* individual each. St_At4 linked to Vm_At4 was found slightly more frequently, namely in 2 *H. obsoletus* and in one *H. obsoletus* transmission to *Catharanthus roseus*. St_At6 and St_At7 were found several times in grapevine samples (15 and 9 %, respectively), but also occurred infrequently in *H. obsoletus* infected *Catharanthus roseus* and in *A. ribauti* infected *Catharanthus roseus*. Both ‘*Ca*. P. solani’ positive *Convolvulus arvensis* samples contained St_At6 and both R. panzeri St_At7. St_At8 and St_At9 were occasionally found in grapevine only. St_At6—St_At9 were all linked to the tuf b1 genotype, but a linkage to the *vmp1* types was not that clear. Vm_At6 was not found in grapevine, but only in *H. obsoletus* or *A. ribauti* infected *Catharanthus roseus*. Vm_At7 came from a single *H. obsoletus* transmission. Vm_At8 was more frequent, it was found in a few grapevines and in *Convolvulus arvensis*. While Vm_At9 originated from a single grapevine, Vm_At10 and Vm_At11 were detected in several grapevine plants and Vm_At11 also in *R. panzeri* and an *A. ribauti* infected *Catharanthus roseus*. Figure 6 shows the phylogenetic ML trees of *stamp* and *vmp1*. As with *secY*, annotated nettle derived samples associated to tuf a and tuf b2 were localised in one branch of the trees. Austrian strains with tuf b1genotypes were found in the majority of the different branches of both the *stamp* (Fig. 6a) and *vmp1* trees (Fig. 6b).

**Table 2:** Genotypes of ‘*Candidatus* Phytoplasma solani’ and their corresponding marker gene annotation. Representative strains/samples for each host plant and the corresponding collection sites are indicated. Strain abbreviations show the host name, the sampling year and a sampling number. Vv: grapevine. Ud: *U. dioica*. Ca: *C. arvensis*. Ho: *H. obsoletus*, Rp: *R. panzeri*. Ar: *A. ribauti*, Cr: *C. roseus*. NÖ- Lower Austria, STMK- Styria, BGLD-Burgenland

Genotype	tuf	vmp1	stamp	secY sequ	secY_ Hinf1	Strain/Sample	Collection site
CPsM4_At1	tuf b2	Vm_At1	St_At1	Se_At1	SecY 2	Vv12_273; Ho12_486; CrHo12_650; Ud13_720	all Rust, BGLD
CPsM4_At2	tuf b2	Vm_At2	St_At2	Se_At1	SecY 2	Vv12_274	Rust, BGLD
CPsM4_At3	tuf a	Vm_At3	St_At3	Se_At1	SecY 2	Ho13_1006	Rust, BGLD
CPsM4_At4	tuf a	Vm_At4	St_At4	Se_At2	SecY 2	Ho13_936; CrHo13_1183	all Einöd, STMK
CPsM4_At5	tuf a	Vm_At5	St_At5	Se_At3	SecY 2	Ho13_838	Rust, BGLD
CPsM4_At6	tuf b1	Vm_At6	St_At6	Se_At5	SecY 1	CrHo12_601; CrAr12_722_1	Falkenstein, NÖ; Langenzersdorf, NÖ
CPsM4_At7	tuf b1	Vm_At8	St_At6	Se_At5	SecY 1	Ca13_RF; Vv12_Kn2	Rust, BGLD; Klosterneuburg, NÖ
CPsM4_At8	tuf b1	Vm_At10	St_At6	Se_At5	SecY 1	Vv12_754	Falkenstein, NÖ
CPsM4_At9	tuf b1	Vm_At11	St_At6	Se_At5	SecY 1	Vv12_753	Falkenstein, NÖ
CPsM4_At10	tuf b1	Vm_At7	St_At7	Se_At5	SecY 1	CrHo12_721	Falkenstein, NÖ
CPsM4_At11	tuf b1	Vm_At9	St_At7	Se_At5	SecY 1	Vv12_Ill6	Illmitz, BGLD
CPsM4_At12	tuf b1	Vm_At11	St_At7	Se_At5	SecY 1	Vv12_752; Rp13_700; CrAr12_722_2	Falkenstein, NÖ; Falkenstein, NÖ; Langenzersdorf, NÖ
CPsM4_At13	tuf b1	Vm_At10	St_At8	Se_At5	SecY 1	Vv12_751	Falkenstein, NÖ
CPsM4_At14	tuf b1	Vm_At10	St_At9	Se_At4	SecY 1	Vv12_Kn6	Klosterneuburg, NÖ

### Occurrence of strains

**Fig. 7:**
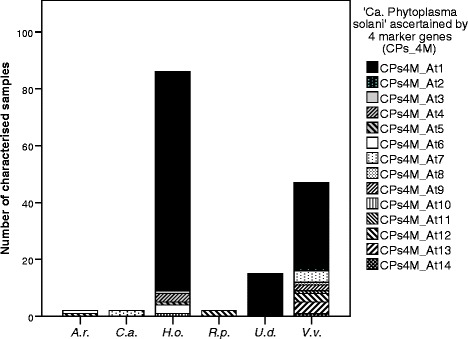
Occurrence of ‘*Ca.* P. solani’ strains in different hosts V.v: grapevine. *U.d. U. dioica*; *C.a. Convolvulus arvensis; H.o. H. obsoletus*, all infected *H. obsoletus* collected from nettle. ‘*Ca.* P.solani’ genotypes either directly determined or in *Catharanthus roseus* after *H. obsoletus* transmission; *R.p. R. panzeri*; *A.r. A. ribauti*, determined or in *Catharanthus roseus* after *A ribauti* transmission. Genotypes were defined on basis of four sequenced marker genes. Genotype definition and database entries are shown in Figs. 5 and 6 and Table 2.

Multilocus sequencing with four markers allows the discrimination of 14 different stolbur genotypes designated as CPsM4_At1—14. Table 2 shows the marker gene composition for each stolbur genotype and representative strains from each host, where the genotype has been found. Figure 7 presents the genotype distribution based on the four markers in the different investigated hosts. The prevalent strain CPsM4_At1 was found in 100 % of the 13 stolbur positive *U. dioica* samples, in 90 % of the stolbur positive *H. obsoletus* samples and in 64 % of the BN infected vines. Strain CPsM4_At2 found in a single grapevine sample in Rust had *tuf* and *secY* sequences identical to CPsM4_At1, but differed in *vmp1* and *stamp*. Strains CPsM4_At3-5 were tuf a strains with different *secY, stamp* and *vmp1* composition and were found only in *H. obsoletus* or *H. obsoletus* infected *Catharanthus roseus*. A more complex situation was identified for the tuf b1 strains. Here various combinations of *stamp* and *vmp1* marker genes were found resulting in 9 different genotype combinations. The most common types were CPsM4_At7 and CPsM4_At13, with the former, being found in bindweeds and in 9 % of the grapevine samples and the latter, being found in 9 % of the grapevine samples. CPsM4_At12 was observed in 6 % of the grapevine samples, in 2 positive *R. panzeri* samples and in one *Catharanthus roseus* infected by *A. ribauti*, while the remaining tuf b1 genotypes were solely ascertained either in a few grapevines or in *Catharanthus roseus* infected by *A. ribauti* or *H. obsoletus*.

## Discussion

The data obtained in the present study provide an insight into the current epidemiology of BN in Austria. Significant numbers of *H. obsoletus* were ascertained at many test sites on *U. dioica* in Lower Austria, Syria and Burgenland (Table 1). In Einöd/Kitzeck, Rust and Falkenstein mass occurrences were observed. Insect densities on nettles and observations on spatial distributions of nettles and insects allowed the conclusion that these populations developed on *U. dioica*. On the contrary, indications for *H. obsoletus* populations developing on bindweed were rare. Only in Klosterneuburg insect catches from bindweed and a clear spatial separation of bindweed and nettle plots allowed the presumption that oviposition and development of larval stages had taken place on bindweed. Total annual catches per year at this site, however, were below 10 specimens (three sampling dates from middle of June till end of July - data not shown) both in 2012 and in 2013. *H. obsoletus* feeding on *Convolvulus arvensis* were also observed in Rust and Kitzeck. But at these locations extremely high population densities on *U. dioica* also occurred. Therefore, a clear allocation of these insects to *Convolvulus arvensis* as host for oviposition and larval development was not possible.

Altogether ‘*Ca*. P. solani’ was ascertained in 25 % of the analysed *H. obsoletus* and in 11 % of the analysed nettles. The analyses ascertained a high level of agreement between phytoplasma presence in nettles and in the *H. obsoletus* collected from them. At collection sites with infected nettles percentages of phytoplasma infected insects ranged between 10 and 38 % (Figs. 1 and 2). Our transmission experiments proved that the insects collected from infected nettles e.g. in Falkenstein 2, Klein Schweinbarth, Einöd/Kitzeck 2 or Rust efficiently infected new host plants. Over 46 % of the nettle plants and more than 55 % of the *Catharanthus roseus* seedlings in these trials became infected.

Although all ‘*Ca*. P. solani’ infected *H. obsoletus* individuals found in this study had been captured on nettles characterization of phytoplasma strains not only resulted in the nettle associated strains CPs4M_At 1-5. In few *Catharanthus roseus* plants from transmission experiments with *H.obsoletus* the bindweed strains CPs4M_At 6 and 10 were also ascertained (Fig. 3, Table 2). In our study the latter strains were never detected in nettle. It therefore seems likely that some insects captured on nettle had migrated there from other host plants.

Interestingly, in our transmission experiments we obtained no phytoplasma infected *Convolvulus arvensis*. Whether this finding is due to the host preference of the insects in the transmission experiments or whether *Convolvulus arvensis* is not a suitable host for the prevalent ‘*Ca*. P. solani’ subtype CPsM4_At1 remains open. All in all the field data obtained in this work allow the conclusion that currently the main epidemiogical cycle of BN includes *H. obsoletus* as insect vector and *U. dioica* as weedy phytoplasma source. This hypothesis is supported by molecular data, as genotype CPsM4_At1 was the most prevalent type found both in grapevine and *H. obsoletus* and was the only genotype detected in nettle.

Our data also indicate a significant change in the epidemiology of BN in Austria within a few years. From 2003 to 2008 noteworthy *H. obsoletus* populations were only observed in Styria in Einöd/Kitzeck and Deutsch Haseldorf/Klöch. Catches at these two sites never exceeded 5 individuals per m^2^ of ground cover. Nettles and bindweeds were sampled at several sites in Lower Austria, Burgenland and Styria. Infected nettles were only found once in 2007 in Einöd/Kitzeck. On the contrary numerous infected bindweeds were observed in all provinces (Riedle-Bauer et al. 2006, Tiefenbrunner et al. 2007, Riedle-Bauer et al. 2008, Sára and Riedle-Bauer 2009). The reasons for these significant changes are so far unclear, but sudden unexpected increases of *H. obsoletus* populations exploiting nettles have previously been observed in other countries, e.g. Germany and South Moravia (Maixner 2011, Šafářova et al. 2011).

In contrast to previous experiments (Riedle-Bauer et al. 2008) in this study we have not been able to find ‘*Ca*. P. solani’ directly in *A. ribauti* individuals, but the insect species transmitted the phytoplasma to *Catharanthus roseus*. Investigations in South Moravia carried out in 2010 and 2011 ascertained the presence of ‘*Ca*. P. solani’ in *A. ribauti* with infection rates ranging between 20 and 33 % (Šafářova et al. 2013). In all our experiments, however, *A. ribauti* was always trapped on *Convolvulus arvensis*, never on *U. dioica*. Moreover, the ability of *A. ribauti* to transmit BN has not been proven, up to now only transmissions to herbaceous hosts have been observed. Therefore it seems unlikely that *A. ribauti* has a significant influence on the current BN epidemiology in Austria. This is supported by genotyping of *Catharanthus roseus* infected in transmission trials with *A. ribauti*, which revealed the presence of stolbur types CPsM4_At6 and CPsM4_At12, but did not provide evidence for the presence of the major genotype of the current epidemic, CPsM4_At1. The CPsM4_At12 genotype was also found occasionally in grapevines. CPsM4_At 6 was also detected infrequently (3 %) in *H. obsoletus*. CPsM4_At6 was not ascertained in grapevine samples, but CPsM4_At9 found in 2 grapevines differed from CPsM4_At6 only in a single point mutation in the highly variable *vmp1*. The occurrence of these and closely related types of ‘*Ca*. Phytoplasma solani’ found both in grapevine and *A. ribauti* gives rise to the possibility that *A. ribauti* might occasionally transmit BN.

In the current analyses we also ascertained *Ca*. ‘P. solani’ in *R. panzeri*. Due to the low number of trapped individuals we only carried out two transmission trials with few insects in which the phytoplasma was not transmitted. Thus further experiments are required to estimate the role of *R. panzeri* as BN vector in Austria. As in *A. ribauti*, the ‘*Ca*. P. solani’ genotype found in *R. panzeri* was not the major type CPsM4_At1, but CPsM4_At12. It is noteworthy that this genotype was found in two grapevines close to the *R. panzeri* collection site. The actual role of *R. panzeri* in the transmission of the more rare BN types in Austria remains to be seen, but recent investigations have not only shown the potential of *R. panzeri* to transmit BN to grapevine in the Banat viticulture region in Serbia, but also revealed that one and the same ‘*Ca*. P. solani’ genotype STOLg dominated in *R. panzeri* and in grapevine in this region (Cvrković et al. 2014). Remarkably, the genotype Rqg50g found in Serbia in *R. panzeri* and in another *Reptalus* species, *R. quinquecostatus*, was identical to CPsM4_At12 (Fig. 6; Cvrković et al. 2014). Rqg50g and the major type found in that study, STOLg clustered with *stamp* types St_At7 (Rqg50g) and St_At8 (STOLg), respectively. The *secY* sequences are identical to Se_A5 and correspond to the virtual Hinf1 RFLP fragments SecY 2 indicating that very closely related genotypes of ‘*Ca*. P. solani’ occur in grapevine and in *Reptalus* species in Austria and Serbia.

As described previously ‘*Ca*. P. solani’ strains can clearly be separated into nettle associated genotypes and genotypes associated with bindweed and other (weedy) plants (Johannesen et al. 2012). This seems to hold true also for the Austrian collection showing a clear separation based on *secY, stamp* and *vmp1* genes for nettle associated strains, which either have be found also in nettle (CPsM4_At1) or with identical sequences to nettle collections (CPsM4_At3-5; Johannesen et al. 2012; CPsM4_At2 has been found only in a single grapevine plant). However, a substitution in the HpaII recognition site in the predominant *tuf* type tuf b2 compared to tuf a is the cause for a tuf b HpaII pattern of the most common genotype CPsM4_At1. Remarkably the type tuf b2 shares a second mutation with tuf a as opposed to tuf b1 (the “classical “tuf b). This substitution on position 727 after the start ATG results in a potentially more stable amino acid substitution from valine in tuf a and tuf b2 to isoleucine in tuf b1 (Fig. 4a).

The *secY* types Se_At4 and Se_At5 separated clearly from the Se_At1-3 associated with nettle. This separation has been found not only in our collections, but also in the analysis of French, German, Italian, Slovenian/Croatian and Swiss secY samples (Johannesen et al., 2012). Se_At5 is identical to SecY D and the *secY* sequences Char1, Moliere, Stol from France and Serbia and all these sequences are associated with tuf b types (Cimerman et al. 2009; Fabre et al. 2011A; 2011B; Johannesen et al. 2012). The difference between Se_At4 and Se_At5 and Se_At1-3 comprised also a change in a Hinf1 site which was used for differentiation by RFLP as shown in Fig. 5. This mutation was accompanied by an amino acid change from aspartate in Se_At4 and Se_At5 to valine in Se_At1-3. It remains to be seen if this RFLP separation between nettle and bindweed/other weeds type is more stable than the previously described *tuf* pattern due to an underlying amino acid exchange and if the differentiation holds true for additional samples apart from current nettle collection in the database and our own samples.

The genes encoding for the surface proteins VMP1 and STAMP are more variable than *secY* or *tuf* (Cimerman et al. 2009; Fabre et al. 2011A). As previously reported for *stamp* (Cvrković et al. 2014; Fabre et al. 2011A; 2011B) and *vmp1* (Johannesen et al. 2012), (Fig. 6) also in the present study nettle associated genotypes (tuf a and tuf b2) forming distinct monophyletic clusters in comparison to polyphyletic tuf b2 (“classical” tuf b) were observed. In addition, our data proved that the nettle associated phenotypes form a *secY* cluster, yielding the RFLP pattern SecY 1 after digestion with Hinf1 (Fig. 5). In the frame of this work 9 different *stamp* and 11 different *vmp1* genotypes could be characterized allowing altogether a discrimination of 14 different ‘*Ca*. Phytoplasma solani’ strains in Austria. Interestingly, the vast majority of the positive samples with more than 90 % of all *H. obsoletus*, 66 % of the grapevines and all nettle samples belonged solely to CPsM4_At1 corresponding to St_At1 and Vm_At1. The bigger part of the variation lied rather in the more rare genotypes of stolbur. A very high prevalence of specific *vmp1* and *stamp* genotypes within the tuf a group have been also described for Germany (for St6/VN1) and to a lesser extent in Croatia/Slovenia (for ST23/VN3; Johannesen et al. 2012). Less clear separation to common types in grapevine and herbal hosts have been described in various regions of Italy based on *vmp1* restriction analysis with Rsa1 (Murolo et al. 2010; Murolo et al. 2013).

Our investigations before 2008 revealed rare infections of nettles and low population densities of *H. obsoletus* in most parts of the country. Together with the prevalent occurrence of CPsM4_At1 in *H. obsoletus* and the sole observation of this type in nettle, it is possible that a sudden range expansion of a certain *H. obsoletus* population feeding on nettle and infected with CPsM4_At1 subtype accounts for the current epidemiology of BN in Austria. A possible explanation for the sole presence of CPsM4_At1 in this plant species might be that before this range expansion nettles were broadly free of ‘*Ca*. P. solani’ leaving room for multiplication of a single ‘*Ca*. P. solani’ stolbur type. A sudden increase of a previously insignificant and therefore undetected tuf b2 transmitting *H. obsoletus* population, however, is equally conceivable.

For the current dispersal of BN in Austria the conclusion can be drawn that the major type of BN is very likely to derive from nettle via infections by *H. obsoletus*. However, the question arises, where the remaining third of infections in grapevine comes from. Partly this might be explained by other, more rare genotypes occurring in *H. obsoletus* related to CPsM4_At6 and CPsM4_At10, possibly derived from infected bindweeds, as it has been described for similar BN genotypes (Langer and Maixner 2004, Johannesen et al. 2012). The existence of a up to now unidentified additional minor host plant can also not be excluded as only 2 of the 90 bindweeds analysed in our study were infected with ‘*Ca*. P. solani’. Potentially, also other vectors such as *R. panzeri* and *A. ribauti* play a role in the dispersal of the less common BN types as the genotypes CPsM4_At6 and CPsM4_At12 were found in these vectors, in vector infected *Catharanthus roseus* and in grapevine. It is also conceivable that infected grapevines are reminiscences of previous infection cycles. Indeed, unlike our current results, in previous years (2003–2008) infected bindweeds were observed in all Austrian vine growing provinces (Riedle-Bauer et al. 2006, Tiefenbrunner et al. 2007, Riedle-Bauer et al. 2008, Sára and Riedle-Bauer 2009) and BN is a disease characterised by sudden outbreaks and subsequent decreases (Maixner, 2011).

It will be also noteworthy to observe if genotypes at the moment only ascertained in *H. obsoletus* (CPsM4_At3-5) will play a role for future grapevine infections. These genotypes have been described in Italy and Slovenia (Johannesen et al. 2012) and CPsM4_At3 (VN3, ST23) has been the most common tuf a type in this study in Slovenia, possibly indicating the potential of this genotype for further spread.

The data obtained in the present study show that in the future the allocation of BN types to epidemiologically relevant host plants cannot be accomplished by PCR/RFLP analysis of the *tuf* gene alone. As previously described (Langer and Maixner, 2004) this procedure allows discrimination between tuf a and tuf b type ‘*Ca*. P. solani’ strains. It also allows the discrimination between ‘*Ca*. P. solani’ and ‘*Ca*. P. convolvuli’, which might also be desirable as our visual observations showed that both phytoplasmas induce comparable symptoms in bindweed. For a clear discrimination between tuf type b1 and tuf type b2 or tuf a, however, a sequence analysis of the *tuf* gene is necessary. Alternatively, PCR/RFLP analysis of the *secY* gene using the endonuclease Hinf1 resulting in *secY* types SecY 2 and SecY 1, respectively and/or multilocus sequencing allow a clearer description of ‘*Ca*. P. solani’ strains
